# Elevation in white blood cell count and development of hyper LDL cholesterolemia

**DOI:** 10.1038/s41598-023-35436-6

**Published:** 2023-05-22

**Authors:** Shota Okutsu, Yoshifumi Kato, Hiroaki Takeoka, Shunsuke Funakoshi, Toshiki Maeda, Chikara Yoshimura, Miki Kawazoe, Atsushi Satoh, Kazuhiro Tada, Koji Takahashi, Kenji Ito, Tetsuhiko Yasuno, Hideyuki Fujii, Shigeaki Mukoubara, Keijiro Saku, Shohta Kodama, Daiji Kawanami, Kosuke Masutani, Hisatomi Arima, Shigeki Nabeshima

**Affiliations:** 1 Department of General Medicine, Faculty of Medicine, Fukuoka University School of Medicine, Fukuoka, Japan; 2 Department of Preventive Medicine and Public Health, Fukuoka University School of Medicine, Fukuoka, Japan; 3 Division of Nephrology and Rheumatology, Department of Internal Medicine, Faculty of Medicine, Fukuoka University School of Medicine, Fukuoka, Japan; 4 Department of Endocrinology and Diabetes Mellitus, Fukuoka University School of Medicine, Fukuoka, Japan; 5 Nagasaki Prefecture Iki Hospital, Nagasaki, Japan; 6 Department of Cardiology, Fukuoka University School of Medicine, Fukuoka, Japan; 7 Department of Regenerative Medicine and Transplantation, Faculty of Medicine, Fukuoka University School of Medicine, Fukuoka, Japan

**Keywords:** Endocrinology, Medical research, Risk factors

## Abstract

To investigate the relationship between white blood cell (WBC) count and incidence of hyper-low-density lipoprotein (LDL) cholesterolemia in a population-based longitudinal study. This is a retrospective study using data of annual health check-ups for residents of Iki City, Japan. A total of 3312 residents (≥ 30 years) without hyper-LDL cholesterolemia at baseline were included in this analysis. Primary outcome was incidence of hyper-LDL cholesterolemia (LDL cholesterol levels ≥ 3.62 mmol/L and/or use of lipid lowering drugs). During follow-up (average 4.6 years), 698 participants development of hyper-LDL cholesterolemia (incidence 46.8 per 1000 person-years). Higher incidence of hyper-LDL cholesterolemia was observed among participants with higher leukocyte count (1st quartile group: 38.5, 2nd quartile group: 47.7, 3rd quartile group: 47.3, and 4th quartile group: 52.4 per 1,000 person-years, *P* = 0.012 for trend). Statistically significant relation was observed even after adjustment for age, gender, smoking, alcohol intake, leisure-time exercise, obesity, hypertension and diabetes: hazard ratio 1.24 (95% confidence interval 0.99 to 1.54) for 2nd quartile group, 1.29 (1.03–1.62) for 3rd quartile group and 1.39 (1.10–1.75) for 4th quartile group, compared with 1st quartile group (*P* for trend = 0.006). Increased WBC count was related to incidence of hyper-LDL cholesterolemia in general Japanese population.

## Introduction

Cardiovascular disease (CVD) is a major cause of death in Japan as well as globally: approximately 18 million people (one-third of total deaths) die of CVD in the world^1^. Increased risks of CVD have been reported to be related to lifestyle-related factors (e.g., smoking, unhealthy dietary habits, physical inactivity and unfavorable metabolic profile [obesity, dyslipidemia, hypertension, and diabetes])^2,3^. Of these, high low-density lipoprotein (LDL) cholesterol levels have been shown to be an established risk factor of CVD^4^. Strategies to lower LDL cholesterol levels and to reduce the risks of CVD need up-to-date knowledge on predictors of future elevation of LDL cholesterol levels. Risk factors for elevation of LDL cholesterol have been shown to involve excessive intake of trans fat, physical inactivity, weight gain, aging, gender, and genetic factors^3,5^. Recently, a few epidemiological studies suggested the association between white blood cell (WBC) count and incidence of hyper-LDL cholesterolemia although the association has not been clearly confirmed^6,7^. The objective was to evaluate the association between increase in WBC count and future incidence of hyper-LDL cholesterolemia in general Japanese population.

## Material and methods

### Study design, participants, and follow-up assessments

The ISSA-CKD (Iki Epidemiological Study of Atherosclerosis and Chronic Kidney Disease) is a retrospective cohort study of residents of the Iki City, Nagasaki Prefecture, Japan. The detailed study design was reported previously^5,8–12^. The Iki Island with approximately 27,000 residents is located in the northern part of Nagasaki Prefecture. Residents of the Iki City (aged ≥ 30 years) are annually invited to health check-ups. From 2008 to 2017, 7895 residents participated in the examinations at least once. After exclusion of 1879 residents who attended only one examination, 2289 with baseline hyper-LDL cholesterolemia (LDL cholesterol levels ≥ 3.62 mmol/L and/or use of lipid lowering drugs), and 415 with missing WBC counts, 3312 individuals were included in the present analysis. This study was conducted in accordance with the Ethical Guidelines for Life Science, Medical and Health Research Involving Human Subjects (Ministry of Education, Culture, Sports, Science and Technology, Ministry of Health, Labour and Welfare, and Ministry of Economy, Trade and Industry, Japan) and the Declaration of Helsinki. Informed consent was obtained using opt-out approach. The study was approved by the Fukuoka University Medical Ethics Review Board (approval number: 2017M010).

### Data collection

At baseline, casual blood samples were collected. All blood tests were conducted measured in a single laboratory (CRC Co., Ltd., Fukuoka, Japan) using standardized procedures throughout the study period. The WBC count was obtained using an automatic blood cell calculator (XN-1000®; Sysmex Corporation, Kobe, Japan). Participants were categorized into 4 groups using quartiles of the leucocyte count: quartile 1 (< 4400/μL), quartile 2 (4400–5300/μL), quartile 3 (5400–6500/μL), and quartile 4 (6500/μL <). Serum LDL cholesterol, High-density lipoprotein (HDL) cholesterol and triglyceride (TG) levels were estimated enzymatically. Plasma glucose concentrations were also estimated enzymatically. HbA1c levels (NGSP [National Glycohemoglobin Standardization Program] value) were estimated using HPLC (high-performance liquid chromatography) method. The presence of diabetes was defined by glucose concentration in fasting status ≥ 7.0 mmol/L, glucose concentration in non-fasting status ≥ 11.1 mmol/L, HbA1c ≥ 6.5%, and/or the use of glucose lowering treatment^13^. Information on smoking, daily alcohol intake, regular exercise habits, and use of medications was obtained using standardized questionnaire. Current smokers were defined as residents who had smoked continuously for more than 6 months or those who had smoked more than one-hundred cigarettes at baseline. Alcohol drinking habits were defined as current daily drinkers or the others. Regular exercise habits were regarded as leisure-time exercise (≥ 30 min) for twice or more per week. Weight and height were measured with light clothes without shoes. Body mass index (BMI [kg/m^2^]) was calculated as weight (kg)/height (m)^2^. Obesity was defined as BMI ≥ 25 kg/m^2^^14^. Blood pressure (BP) was measured twice in a sitting position, in the right upper arm, using cuff with appropriate size, using mercury, automatic or aneroid sphygmomanometer, after at least 5 min of rest, by trained staff, in accordance with standardized guideline^15^. The average value of the two BP values was used in the analysis. Hypertension was defined as BP ≥ 140/90 mmHg, and/or use of BP lowering drugs^16^.

### Definition of outcome

During the study period between 2008 and 2017, the first visit of the health examination for each participant was defined as baseline examination. Participants were followed-up to the last visit of the follow-up health examination. The endpoint was incidence of hyper-LDL cholesterolemia (elevation of LDL cholesterol level to ≥ 3.62 mmol/L and/or initiation of lipid lowering agents during follow-up)^17^. Participants whose LDL cholesterol levels returned to < 3.62 mmol/L without lipid lowering agents at the last follow-up visit were not regarded as incident hyper-LDL cholesterolemia. We have also conducted sensitivity analysis defining the first time diagnosed with hyper-LDL cholesterolemia as incidence.

### Statistical analysis

Continuous characteristic values were shown as mean ± SD, and *p* values for trend across WBC count groups were estimated with use of simple regression models. Categorical characteristic variables were shown as participant number (%), and *p* values for trends across WBC count groups were estimated with use of logistic regression models. With regard to incidence of hyper-LDL cholesterolemia in each WBC group, the person-year method was used. The association between WBC count groups and incidence of hyper-LDL cholesterolemia was investigated using Cox’s proportional hazards analysis, and the effects of WBC count were reported using hazard ratios (HRs) and 95% confidence intervals (95% CIs). Proportional hazards assumption was assessed using cumulative sums of martingale-based residuals and using Schoenfeld residuals^18^. Multivariable-adjusted Cox’s proportional hazards included age, gender, current smoking habits, current alcohol intake, exercise habits, obesity, hypertension, and diabetes as covariates. *P* values for trend across 4 groups of WBC count were obtained from tests for the presence of an association between an ordinal variable of WBC count with 4 categories and the outcome using Cox’s proportional hazards models. Subgroup analyses were conducted according to other risk factors such as age, gender, smoking, exercise, obesity, diabetes, and the differences in the associations between WBC count and incidence of the outcome between subgroups were tested by adding an interaction term to the statistical model. *P* < 0.05 was considered statistically significant. SAS version 9.4 was used for the statistical analysis of the paper.

### Ethics approval

The study was conducted according to the guidelines of the Declaration of Helsinki of 1975, revised in 2013, and approved by Fukuoka University Clinical Research and Ethics Centre (No.2017M010).

## Results

Baseline characteristics by quartiles of leucocyte count were shown in Table 1. Higher leucocyte counts were associated with younger age and male gender. Participants with higher leucocyte counts were more likely to be current smokers, current drinkers, obese, hypertensive and diabetic.

**Table 1:** Characteristic of the participants according to leucocyte count.

	Quartile of white blood cell count (/μL)				*P* trend
	< 4500 (N = 847)	4500–5300 (N = 839)	5400–6500 (N = 819)	> 6500 (N = 807)	
Age, years	60.1 ± 10.7	59.5 ± 11.0	58.8 ± 11.4	57.1 ± 12.0	< .0001
Female	543 (64.1)	413 (49.2)	336 (41.0)	309 (38.3)	< .0001
Smoking	74 (8.7)	128 (15.3)	179 (21.9)	352 (43.6)	< .0001
Current daily alcohol intake	171 (20.6)	233 (28.0)	253 (31.2)	270 (33.7)	< .0001
Regular Exercise	223 (27.1)	232 (28.0)	206 (25.6)	201 (25.2)	0.23
Obesity	157 (18.5)	231 (27.5)	259 (31.6)	267 (33.1)	< .0001
Body mass index, kg/m^2^	22.5 ± 3.10	23.3 ± 3.29	23.6 ± 3.48	23.9 ± 3.60	< .0001
Systolic blood pressure, mmHg	127.1 ± 18.6	128.5 ± 18.7	128.5 ± 18.7	129.3 ± 19.2	< .0001
Diastolic blood pressure. mean, mmHg	73.4 ± 10.8	74.8 ± 11.1	74.4 ± 11.1	75.9 ± 11.6	< .0001
Hypertension	309 (36.5)	348 (41.5)	353 (43.1)	350 (43.4)	0.003
HbA1c %	5.30 ± 0.50	5.35 ± 0.60	5.45 ± 0.86	5.43 ± 0.72	< .0001
Diabetes mellitus	42 (4.96)	54 (6.44)	72 (8.79)	79 (9.79)	< .0001
High-density lipoprotein cholesterol, mmol/dL	1.69 ± 0.43	1.62 ± 0.45	1.59 ± 0.43	1.51 ± 0.40	< .0001
Low-density lipoprotein cholesterol, mmol/dL	2.76 ± 0.53	2.80 ± 0.54	2.82 ± 0.53	2.80 ± 0.55	0.14
Triglyceride, mmol/dL	1.04 ± 0.66	1.22 ± 0.90	1.29 ± 0.95	1.56 ± 1.31	< .0001

Values are mean ± SD or N(%).

During 4.6 years of mean follow-up, incidence of hyper-LDL cholesterolemia was observed among 698 participants (incidence 46.8/1000 person-years). Table 2 shows the association between leucocyte count and incidence of hyper- LDL cholesterolemia. The annual incidence of hyper-LDL cholesterolemia increased with elevation of the WBC count (38.5 per 1000 person-years in quartile 1, 47.7 in quartile 2, 47.3 in quartile 3 and 52.4 in quartile 4) (*P* = 0.012 for trend). There was no evidence that proportional hazards assumption was violated across quartile groups of WBC count. Significant associations were also observed after control of the confounding effects of other risk factors including age, gender, current smoking, current drinking, regular exercise, obesity, hypertension, and diabetes (HRs 1.24 [95% CI 0.99–1.54] for quartile 2, 1.29 [1.03–1.62] for quartile 3, 1.39 [1.10–1.75] for quartile 4 compared with quartile 1) (*P* = 0.006 for trend). Comparable associations were obtained from sensitivity analysis defining the first time diagnosed with hyper-LDL cholesterolemia as incidence (Supplementary Table 1).

**Table 2:** Risks of hyper-low-density lipoprotein cholesterolemia according to quartiles of the white blood cell count.

	Quartile of white blood cell count (/μL)				*P* trend
	< 4500 (N = 847)	4500–5300 (N = 839)	5400–6500 (N = 819)	> 6500 (N = 807)	
N of event/person-years	153/3970	185/3879	173/3655	187/3569	
Incidence rate (/1000 person-years)	38.5	47.7	47.3	52.4	
Crude hazard ratio (95% CI)	1.0 (Reference)	1.25 (1.01–1.55)	1.22 (0.977–1.51)	1.35 (1.09–1.67)	0.012
Multivariable-adjusted* hazard ratio (95% CI)	1.0 (Reference)	1.24 (0.99–1.54)	1.29 (1.03–1.62)	1.39 (1.10–1.75)	0.006

*Adjusted for age, sex, smoking, daily drinking, exercise, obesity, hypertension, and diabetes. CI: Confidence interval.

Table 3 shows the association between quartiles of white blood cell count and hyper-low-density lipoprotein cholesterolemia in subgroups. Comparable effects of leucocyte count for incidence of hyper-LDL cholesterolemia were observed between subgroups defined by age (< 65 vs. ≥ 65 years), gender, smoking, exercise, obesity, and diabetes (all *p* interaction > 0.1).

**Table 3:** Association between quartiles of white blood cell count and hyper-low-density lipoprotein cholesterolemia in subgroups.

		Quartile of white blood cell count (/μL)				*P* for interaction
		< 4500 (N = 847)	4500–5300 (N = 839)	5400–6500 (N = 819)	> 6500 (N = 807)	
Age						
< 65 years	N of event	83	121	110	121	
	Hazard ratio* (95% CI)	1 (reference)	1.50 (1.13–1.99)	1.48 (1.10–1.98)	1.42 (1.05–1.91)	
≥ 65 years	N of event	70	64	63	66	
	Hazard ratio* (95% CI)	1 (reference)	0.93 (0.66–1.32)	1.06 (0.75–1.52)	1.33 (0.94–1.90)	0.86
Gender						
Male	N of event	40	80	85	105	
	Hazard ratio* (95% CI)	1 (reference)	1.33 (0.91–1.95)	1.30 (0.89–1.89)	1.54 (1.05–2.25)	
Female	N of event	113	105	88	82	
	Hazard ratio* (95% CI)	1 (reference)	1.17 (0.89–1.54)	1.29 (0.97–1.71)	1.21 (0.89–1.64)	0.75
Smoking						
Yes	N of event	13	21	29	87	
	Hazard ratio* (95% CI)	1 (reference)	0.96 (0.48–1.91)	1.01 (0.52–1.96)	1.57 (0.87–2.82)	
No	N of event	140	164	144	100	
	Hazard ratio* (95% CI)	1 (reference)	1.29 (1.02–1.63)	1.36 (1.07–1.73)	1.24 (0.95–1.62)	0.08
Exercise						
Yes	N of event	36	50	45	52	
	Hazard ratio* (95% CI)	1 (reference)	1.35 (0.88–2.08)	1.45 (0.92–2.27)	1.90 (1.21–2.98)	
No	N of event	113	132	128	131	
	Hazard ratio* (95% CI)	1 (reference)	1.19 (0.92–1.55)	1.21 (0.94–1.58)	1.19 (0.91–1.55)	0.39
Obesity						
Yes	N of event	34	62	66	68	
	Hazard ratio* (95% CI)	1 (reference)	1.23 (0.81–1.88)	1.30 (0.81–1.88)	1.22 (0.79–1.87)	
No	N of event	119	123	107	119	
	Hazard ratio* (95% CI)	1 (reference)	1.24 (0.96–1.61)	1.28 (0.98–1.68)	1.49 (1.13–1.96)	0.77
Diabetes						
Yes	N of event	8	15	18	23	
	Hazard ratio* (95% CI)	1 (reference)	1.29 (0.54–3.07)	1.30 (0.60–3.05)	1.38 (0.61–3.12)	
No	N of event	145	170	155	164	
	Hazard ratio* (95% CI)	1 (reference)	1.21 (0.96–1.52)	1.27 (1.00–1.60)	1.34 (1.05–1.70)	0.98

*Adjusted for age (except for subgroup analysis by age), sex (except for subgroup analysis by sex), smoking, exercise, obesity (except for subgroup analysis by obesity), and diabetes.

## Discussion

In our study, we found that there was a statistically significant relationship between the increase in WBC count and future development of hyper-LDL cholesterolemia in general Japanese men and women. This result was also statistically significant after adjusting for age, gender, smoking, drinking, regular exercise, obesity, hypertension, and diabetes. There were also comparable associations between subgroups defined by age, gender, smoking, exercise, obesity, and diabetes.

There are several reports from observational studies which reported the association between white blood cell count and hyper-LDL cholesterolemia. In a cross-sectional study of 10,866 Chinese hypertensive populations, there was a positive correlation between WBC count and serum LDL cholesterol with or without diabetes^7^. At a health care center of a hospital, 2588 Japanese health checkup participants were followed for 4 years, and lymphocyte counts were significantly positively correlated with incidence of hyper-LDL cholesterolemia^6^. In the present long-term, large-scale observational study of general Japanese confirmed the results of the prior studies and have shown that increase in WBC count was associated with future incidence of hyper-LDL cholesterolemia.

Current evidence of the association between WBC count and LDL cholesterol has been mainly derived from Asia (including Japan)^6,7,19^. This fact might in part be attributable to lower levels of inflammatory markers in Asian people^20^, whose dietary pattern is correlated with higher consumption of soybean products, fish, seaweeds, vegetables, fruits and green tea and with lower consumption of meat and fat^21^, than those in Western people. Future research to compare the associations between WBC count and LDL cholesterol across different regions is required to confirm possible regional heterogeneity.

Smoking is a possibly important confounding factor in the present analysis because WBC count is strongly associated with current smoking status^22^. In the present analysis, however, there were no clear evidence of interaction between WBC count and current smoking for incidence of hyper-LDL cholesterolemia (*P* = 0.90 for interaction). Furthermore, in multivariable analysis, the association between WBC count and incidence of hyper-LDL cholesterolemia was independent of other confounding factors including current smoking. Therefore, the relationship between WBC count and hyper-LDL cholesterolemia seems to be independent of possible confounding effects of smoking.

The mechanism of the link between elevated leukocyte count and hyper-LDL cholesterolemia have not been clarified, but the following hypothesis is conceivable. Elevation of leukocytes is likely to represent inflammatory reaction in adipose tissue associated with visceral fat accumulation^23–26^, which has been shown to cause insulin resistance. Insulin resistance has been shown to elevate LDL-cholesterol levels through pool of LDL precursors including very-low-density lipoprotein (VLDL) due to increased production and decreased clearance associated with decreased activity of lipoprotein lipase (LPL)^27,28^. More specifically, insulin resistance has been shown to reduce inhibition of hormone-sensitive lipase in adipose tissue by hyperinsulinemia and increased free fatty acid (FFA) level in hepatocyte^27,28^. Elevated of FFA has been shown to reduce in degradation of apo B in hepatocytes^27,28^. It also suppresses phosphoinositide (PI) 3-kinase mediated apoB degradation and accelerates the action of microsomal triglyceride transfer protein (MTP), a rate-limiting factor of VLDL assembly^27,28^. Moreover, TG-riched and enlarged VLDL1 has been shown to increase by increased activity of two factors involved in the formation of VLDL_1_: phospholipase D1 and ADP ribosylation factor 1 (ARF-1)^27^. Insulin resistance is also likely to increase circulated LDL through LDL receptor (LDLR) degradation due to upregulated hepatic lipase levels and decreased LDLR binding ability due to increase in oxidized, saccharified or small-dense LDL^27,29^. Small-dens LDL has been shown to be created by increased activity of Cholesteryl ester transfer protein (CETP), which transfer TGs from TG-rich lipoproteins to LDL, and hepatic triglyceride lipase^27,28^. Furthermore, hepatic insulin resistance appears to increase proprotein convertase subtilisin/kexin type 9 (PCSK9) levels, which promotes degradation and reduction of LDLR and subsequent increase in LDL^29,30^.

This study has several limitations. First, because this was a retrospective cohort study with irregular visits for some participants, the actual data of the new onset of hyper-LDL cholesterolemia was not clear. Next, health-conscious people were more likely to have visited health examinations and were more likely to have been included in the analysis than residents with unhealthy lifestyle. Third, we have no information on WBC fraction such as neutrophil count, neutrophil/lymphocyte ratio etc., and other inflammatory marker such as C reactive protein, TNF-α, IL-1β etc. Fourth, we do not have information on co morbidities (such as infection, cancer, leukemia etc.) which might affect WBC counts. Fifth, we have missing information on nutritional factors.

## Conclusion

In the general population of Japanese adults, increase in leukocyte count was related to future development of hyper-LDL cholesterolemia. In addition to management of traditional cardiovascular risk factors, high-risk approach based on WBC count might add further protection against the future onset of hyper-LDL cholesterolemia and subsequent cardiovascular events/mortality.

## Supplementary Information

Supplementary Information.

## Data Availability

The datasets generated and/or analysed during the current study are not publicly available due to the property of the Iki CIty, Nagasaki, Japan and cannot be made public as it may result in violation of the Act on the Protection of Personal Information of the Japanese government but are available from the corresponding author on reasonable request.

## References

[1] Roth GA (2017). Global, regional, and national burden of cardiovascular diseases for 10 Causes, 1990 to 2015. J. Am. Coll. Cardiol..

[2] Zmysłowski A, Szterk A (2017). Current knowledge on the mechanism of atherosclerosis and pro-atherosclerotic properties of oxysterols. Lipids Health Dis..

[3] Catapano AL (2016). 2016 ESC/EAS guidelines for the management of dyslipidaemias: The task force for the management of dyslipidaemias of the european society of cardiology (ESC) and european atherosclerosis society (EAS). Eur. Heart J..

[4] Nabel EG, Braunwald E (2012). A tale of coronary artery disease and myocardial infarction. N. Engl. J. Med..

[5] Shota O (2021). Effects of weight gain after 20 years of age and incidence of hyper-LDL cholesterolemia: Iki epidemiological study of atherosclerosis and chronic kidney disease (ISSA-CKD). J. Clin. Med..

[6] Oda E (2014). Longitudinal associations between lymphocyte count and LDL cholesterol in a health screening population. J. Clin. Transl. Endocrinol..

[7] Yanhong L (2017). Association of peripheral differential leukocyte counts with dyslipidemia risk in Chinese patients with hypertension: insight from the China stroke primary prevention trial. J. Lipid. Res..

[8] Ishida S (2021). White blood cell count and incidence of hypertension in the general Japanese population: ISSA-CKD study. PLoS ONE.

[9] Miyabayashi I (2020). Uric acid and prevalence of hypertension in a general population of Japanese: ISSA-CKD Study. J. Clin. Med. Res..

[10] Yasuno T (2020). Effects of HbA1c on the development and progression of chronic kidney disease in elderly and middle-aged Japanese: Iki Epidemiological study of atherosclerosis and chronic kidney disease (ISSA-CKD). Intern..

[11] Maeda T (2019). Usefulness of the blood pressure classification in the new 2017 ACC/AHA hypertension guidelines for the prediction of new-onset chronic kidney disease. J. Hum. Hypertens..

[12] Fujii H (2021). Eating speed and incidence of diabetes in a japanese general population: ISSA-CKD. J. Clin. Med..

[13] Shurraw S, Tonelli M (2011). Alberta kidney disease network: Association between glycemic control and adverse outcomes in people with diabetes mellitus and chronic kidney disease: A population-based cohort study. Arch. Intern..

[14] Examination Committee of Criteria for ‘Obesity Disease’ in Japan: Japan Society for the Study of Obesity New Criteria for “Obesity Disease” in Japan. *Circ. J.* **66**, 987–992 (2002). 10.1253/circj.66.987 12419927

[15] The Japanese Society of Cardiovascular Disease Prevention. *Handbook for Cardiovascular Prevention*. (Hokendojinsha; Tokyo, Japan: 2014).

[16] Umemura S (2019). The japanese society of hypertension guidelines for the management of hypertension (JSH 2019). Hypertens. Res..

[17] Kinoshita M (2018). Japan atherosclerosis society (JAS) guidelines for prevention of atherosclerotic cardiovascular diseases 2017. J. Atheroscler. Thromb..

[18] Lin DY (1993). Checking the cox model with cumulative sums of the martingale-based residuals. Biometrika.

[19] Meng W (2012). Association between leukocyte and metabolic syndrome in urban han chinese: A longitudinal cohort study. PLoS ONE.

[20] Hisatomi A (2008). High-sensitivity c-reactive protein and coronary heart disease in a general population of Japanese: The Hisayama study. Arterioscler. Thromb. Vasc. Biol..

[21] Taichi S (2007). Dietary patterns and cardiovascular disease mortality in Japan: A prospective cohort study. Int. J. Epidemiol..

[22] Pedersen KM (2019). Smoking and Increased white and red blood cells. Arterioscler. Thromb. Vasc. Biol..

[23] Romeo GR, Lee J, Shoelson SE (2012). Metabolic syndrome, insulin resistance, and roles of inflammation–mechanisms and therapeutic targets. Arterioscler. Thromb. Vasc. Biol..

[24] Andersen CJ, Murphy KE, Fernandez ML (2016). Impact of obesity and metabolic syndrome on immunity. Adv. Nutr..

[25] Hagita H, Osaka M, Shimokado K, Yoshida M (2011). Adipose inflammation initiates recruitment of leukocytes to mouse femoral artery: Role of adipo-vascular axis in chronic inflammation. PLoS ONE.

[26] Jung UJ, Choi MS (2014). Obesity and its metabolic complications: The role of adipokines and the relationship between obesity, inflammation, insulin resistance, dyslipidemia and nonalcoholic fatty liver disease. Int. J. Mol. Sci..

[27] Vergès B (2015). Pathophysiology of diabetic dyslipidaemia: Where are we?. Diabetologia.

[28] Hirano T (2018). Pathophysiology of diabetic dyslipidemia. J. Atheroscler. Thromb..

[29] Arvind, A. *et al*. *Lipid and Lipoprotein Metabolism in Liver Disease. MDText.com*, Inc.; 2000-. https://www.ncbi.nlm.nih.gov/books/NBK326742/26561702

[30] Krysa JA, Ooi TC, Proctor SD, Vine DF (2017). Nutritional and lipid modulation of PCSK9: Effects on cardiometabolic risk factors. J. Nutr..

