# Supplementary material for: Elevation in white blood cell count and development of hyper LDL cholesterolemia
Source: Sci Rep. 2023 May 22;13:8292. doi: 10.1038/s41598-023-35436-6 (PMC10202917; doi:10.1038/s41598-023-35436-6)
Supplement: Supplementary file 1 — Supplementary Information. [file 41598_2023_35436_MOESM1_ESM.docx]

**Supplementary Table 1. Risks of hyper-low-density lipoprotein cholesterolemia (defined without confirmation using information of the last follow-up visit) according to quartiles of the white blood cell count**

|  | Quartile of white blood cell count (/μL) | | | |  |
| --- | --- | --- | --- | --- | --- |
|  | <4500  (N=847) | 4500-5300  (N=839) | 5400-6500  (N=819) | >6500  (N=807) | P trend |
| N of event /  person-years | 307/3464 | 343/3366 | 324/3216 | 337/3098 |  |
| Incidence rate  (/1000 person-years) | 89 | 102 | 101 | 109 |  |
| Crude hazard ratio  (95% CI) | 1.0 (Reference) | 1.16 (0.99-1.35) | 1.14 (0.98-1.34) | 1.24 (1.06-1.44) | 0.012 |
| Multivariable-adjusted* hazard ratio  (95% CI) | 1.0 (Reference) | 1.14 (0.97-1.33) | 1.16 (0.99-1.36) | 1.30 (1.10-1.53) | 0.003 |

*Adjusted for age, sex, smoking, daily drinking, exercise, obesity, hypertension, and diabetes. CI, confidence interval
